# Performance of Idylla^™^ *RAS*-*BRAF* mutation test for formalin-fixed paraffin-embedded tissues of colorectal cancer

**DOI:** 10.1007/s10147-022-02167-z

**Published:** 2022-04-26

**Authors:** Yusuke Makutani, Kazuko Sakai, Masahiro Yamada, Toshiaki Wada, Takaaki Chikugo, Takao Satou, Yoko Iwasa, Hidekazu Yamamoto, Marco A. de Velasco, Kazuto Nishio, Junichiro Kawamura

**Affiliations:** 1 Department of Surgery, Faculty of Medicine, Kindai University, Osaka-sayama, Osaka 589-8511 Japan; 2 Department of Genome Biology, Faculty of Medicine, Kindai University, Ohnohigashi 377-2, Osaka-Sayama, Osaka 589-8511 Japan; 3 Department of Surgery, Shiga General Hospital, Moriyama, Shiga 524-8524 Japan; 4 Department of Diagnostic Pathology, Kindai University Hospital, Osaka-Sayama, Osaka 589-8511 Japan; 5 Department of Diagnostic Pathology, Shiga General Hospital, Moriyama, Shiga 524-8524 Japan

**Keywords:** Colorectal cancer, *KRAS* mutation, Anti-EGFR antibody, Companion diagnosis

## Abstract

**Background:**

The Biocartis Idylla^™^ platform is a fully automated, real-time PCR-based diagnostic system. The Idylla^™^ *KRAS* and *NRAS*-*BRAF* Mutation Tests have been developed for the qualitative detection of mutations in *KRAS*, *NRAS* and *BRAF* genes, facilitating the genomic profiling of patients with colorectal cancer. The aim of the present study was to evaluate clinical performances of these tests in Japan.

**Methods:**

The *RAS* and *BRAF* mutation statuses of 253 formalin-fixed paraffin-embedded (FFPE) colorectal cancer tissues were analyzed using the Investigational Use Only Idylla^™^ *KRAS* Mutation Test and the Idylla^™^ *NRAS*-*BRAF* Mutation Test and an in vitro diagnostics (IVD) kit (MEBGEN RASKET^™^-B kit).

**Results:**

The success rate for obtaining a valid mutational data without retest of the Idylla tests was 97.6% (247/253): 111 *KRAS* mutations (43.8%), 9 *NRAS* mutations (3.6%), and 36 *BRAF* V600E mutations (14.2%) were detected using the Idylla tests. Compared with the MEBGEN RASKET-B results, the positive concordance rate was 97.4%, the negative concordance rate was 95.7%, and the overall concordance rate was 95.3% (*κ* = 0.919, 95% CI 0.871–0.967). The average turnaround time to Idylla^™^ *KRAS* and *NRAS*-*BRAF* Mutation Test was 5.6 working days (range: 3–11 days).

**Conclusion:**

This result demonstrates a high concordance between the Idylla^™^ *KRAS* and *NRAS-BRAF* Mutation Tests and an existing IVD kit. In this manner, the Idylla^™^ mutation tests were validated for the detection of clinically significant *KRAS, NRAS, and BRAF* mutations in FFPE samples from colorectal cancer patients.

**Supplementary Information:**

The online version contains supplementary material available at 10.1007/s10147-022-02167-z.

## Introduction

Colorectal carcinoma (CRC) is one of the most common cancers in the world and is a leading cause of cancer mortality in men and women [1]. The epidermal growth factor receptor (EGFR) and its downstream signaling pathways, including the RAS-RAF-MAPK and phosphatidylinositol 3-kinase (PI3K)-Akt pathways, play important roles in tumor growth in CRC [2]. Anti-EGFR antibodies, such as cetuximab and panitumumab, improve the prognosis of patients with metastatic CRC (mCRC) [3, 4]. However, the therapeutic efficacy of anti-EGFR therapy is limited to patients with wild-type (WT) RAS genotypes: mutations in exons 2, 3, and 4 of the *KRAS*, *NRAS*, and exons 15 of the *BRAF* genes confer resistance to these antibodies [3, 4]. The reduced efficacy of anti-EGFR therapy was initially thought to be the result of mutations within *KRAS* exon 2 (codons 12 and 13). Subsequently, through two large clinical trials (PRIME and CRYSTAL), additional mutations that conferred resistance to anti-EGFR therapy were discovered [4]. For example, the *BRAF* V600E mutation was detected in 4–18% of CRC cases and was found to be responsible for a reduced response to EGFR inhibitors and a worse prognosis [5].

Several methods for the detection of *KRAS*, *NRAS*, and *BRAF* mutations in formalin-fixed paraffin-embedded (FFPE) tissues have been reported. These mutation kits have been marketed for colorectal cancer patients. Routine genotyping of DNA extracted from FFPE samples is performed using laboratory-based assays or in vitro diagnostic medical device (IVD) kits. Routine testing methods include Sanger sequencing, pyrosequencing, next-generation sequencing (NGS) [6], immunohistochemistry, and real-time PCR-based testing. Each of these kits has its own assay conditions in terms of sensitivity, specificity, cost, turnaround time, level of automation and multiplexing, special equipment and skilled staff. The turnaround time is a particularly important parameter for patients with rapidly progressing metastases. The turnaround time of the Idylla platform is reported to be shortened to an average of 4.5 working days [7].

The Idylla^™^ system (Biocartis, Mechelen, Belgium) is a fully automated, real-time PCR-based molecular diagnostic system [8–14] and is an example of an in vitro diagnostic medical device (IVD) that can be used for the qualitative detection of mutations. The Idylla^™^ *KRAS* and *NRAS*-*BRAF* Mutation Tests qualitatively detect mutations in *KRAS* codons 12, 13, 59, 61, 117, and 146; *NRAS* codons 12, 13, 59, 61, 117, and 146; and *BRAF* codon 600 in FFPE human malignant CRC tissue. When performed on the Idylla^™^ platform, Idylla^™^ mutation tests are automated sample-to-results solutions that integrate the analytical process into a single cartridge, eliminating the need for time-consuming pretreatment processes, including the steps for FFPE processing. To date, the CE-IVD Idylla^™^ *KRAS* Mutation Test and the Idylla^™^ *NRAS*-*BRAF* Mutation Test have been developed to detect *KRAS*, *NRAS*, and *BRAF* mutations in colorectal cancer patients. However, the Idylla^™^ system has not yet been approved for use in Japan, and a formal clinical performance study conducted in Japan is needed. In the present study, we compared the clinical performances of two Idylla^™^ tests (the Idylla^™^ *KRAS* Mutation Test and the Idylla^™^ *NRAS-BRAF* Mutation Test) used in combination with an IVD kit (MEBGEN RASKET^™^-B kit; Medical & Biological Laboratories Co., Tokyo, Japan) [15, 16] using FFPE tissue samples from colorectal cancer patients.

## Materials and methods

### Samples and study design

A series of 275 archived FFPE tissues from 275 Japanese patients with colorectal cancer diagnosed at the Kindai University Hospital and Shiga General Hospital was obtained. All cases were staged based on the criteria from the Japanese Classification of Colorectal, Appendiceal, and Anal Carcinoma [17]. And were selected by searching through archival material for the years 2017–2021. The 275 cases included 28 *BRAF* V600E positive cases obtained by prescreening 250 cases prior to the study. All the patients enrolled in the study had provided informed consent for the use of their resected tissue samples. This study was approved by the ethical committees of the Kindai University Faculty of Medicine and Shiga General Hospital (Authorization Number: R02-312).

**Fig. 1:**
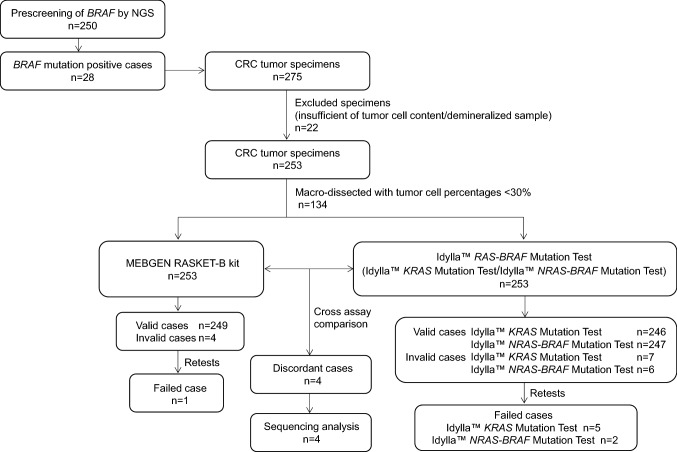
Procedure for enrolling patient samples in the study. The formalin-fixed paraffin-embedded (FFPE) sample preparation algorithm used prior to Idylla^™^ *KRAS* and *NRAS*-*BRAF* mutation testing is shown as a pre-analytical process. Histologically confirmed, retrospectively collected FFPE colorectal cancer tissue samples were identified, and tissue Sects. 5 µm thick were sampled as close as possible to the sections used for reference testing. Prior to the analysis, the tumor contents and area were determined using an H&E-stained slide by a consultant histopathologist at both sites. If required, macro-dissections were performed to ensure a tumor nuclei content of ≥ 30% in each sample used for analysis. Mutations in *KRAS*, *NRAS*, and *BRAF* mutations were analyzed using the Idylla^™^ *KRAS* Mutation Test and the Idylla^™^ *NRAS*-*BRAF* Mutation Test (maximum three slide each). DNA was extracted from individual slides, and the mutations were analyzed using the MEBGEN RASKET^™^-B kit. Concordance between the Idylla results and the MEBGEN RASKET-B results was then determined. Discordant cases were analyzed using amplicon deep sequencing

The study design is summarized in Fig. 1. FFPE specimens from 275 colorectal cancer patients were sliced into 5 μm thin slices. Twenty-two cases were excluded from the study, including 21 cases with a tumor percentage of less than 30% based on hematoxylin and eosin (H&E) staining and one demineralized specimen. A total of 253 cases were included in the study. The tumors areas were marked by pathologists, 134 of 253 specimens were manually dissected, and only specimens with tumor percentages ≥ 30% were subjected to the Idylla^™^ *KRAS* and *NRAS-BRAF* Mutation Tests and the MEBGEN RASKET^™^-B kit.

### Idylla^™^*KRAS* and *NRAS*-*BRAF* mutation tests (Idylla^™^)

The Idylla^™^ *KRAS* Mutation Test is a single-use cartridge-based test designed for the qualitative detection of 21 different mutations. In this study, the Idylla IUO assay was used. The mutations included those in *KRAS* codons 12, 13, 59, 61, 117 and 146. The Idylla^™^ *NRAS*-*BRAF* Mutation Test utilizes PCR reactions to amplify 15 mutations in codons 12, 13, 59, 61, 117, 146 of the *NRAS* oncogene and codon 600 of the *BRAF* oncogene. Table 1 lists the mutations that are detected by the test. Detailed procedures were summarized in Supplementary Method as described previously [18]. The performance evaluation of the Idylla^™^ *KRAS* and *NRAS*-*BRAF* Mutation Test was performed in the Department of Genome Biology, Kindai University Faculty of Medicine.

**Table 1:** *KRAS*, *NRAS,* and *BRAF* mutations detected by the Idylla^™^ *KRAS* and *NRAS-BRAF* mutation test

Gene	Codon (exon)	Amino acid change	Coding region change
*KRAS* mutation detection	Codon 12 (exon 2)	G12C	c.34G>T
		G12R	c.34G>C
		G12S	c.34G>A
		G12A	c.35G>C
		G12D	c.35G>A
		G12V	c.35G>T
	Codon 13 (exon 2)	G13D	c.38G>A
	Codon 59 (exon 3)	A59E	c.176C>A
		A59G	c.176C>G
		A59T	c.175G>A
	Codon 61 (exon 3)	Q61H	c.183A>C; c.183A>T
		Q61K	c.181C>A; c.180_181delinsAA
		Q61L	c.182A>T
		Q61R	c.182A>G
	Codon 117 (exon 4)	K117N	c.351A>C; c.351A>T
	Codon 146 (exon 4)	A146P	c.436G>C
		A146T	c.436G>A
		A146V	c.437C>T
*NRAS* mutation detection	Codon 12 (exon 2)	G12C	c.34G>T
		G12D	c.35G>A
		G12A	c.35G>C
		G12V	c.35G>T
	Codon 13 (exon 2)	G13D	c.38G>A
		G13V	c.38G>T
		G13R	c.37G>C
	Codon 59 (exon 3)	A59T	c.175G>A
	Codon 61 (exon 3)	Q61K	c.181C>A
		Q61L	c.182A>T
		Q61R	c.182A>G
		Q61H	c.183A>C
	Codon 117 (exon 4)	K117N	c.351G>C; c.351G>T
	Codon 146 (exon 4)	A146T	c.436G>A
*BRAF* mutation detection	Codon 600 (exon15)	V600E	c.1799T>A

### MEBGEN RASKET^™^-B kit

Genomic DNA was extracted from FFPE tumor samples using a standard procedure, and 10–20 ng/μl concentrations of DNA were subjected to PCR reactions. In total, 49 *KRAS*, *NRAS*, and *BRAF* mutations were analyzed using the MEBGEN RASKET^™^-B kit [15, 16]; the PCR reaction reverse sequence-specific oligonucleotide method was used for all the samples. Mutations were determined using multiplex PCR and the xMAP^®^ (Luminex^®^) technology. The mutations detected by the MEBGEN RASKET^™^-B kit are listed in Supplementary Method. This procedure was performed in the laboratories of LSI-Medience Co. (Tokyo, Japan) and SRL Inc. (Tokyo, Japan). Finally, all the data were collected and compared.

### Sequencing analysis

DNA were purified with the use of an Allprep DNA/RNA FFPE Kit (Qiagen, Valencia, CA, USA) according to the manufacturer’s instructions. Amplicon sequencing were performed using the Ion AmpliSeq Colon and Lung Cancer Panel (Thermo Fisher Scientific, Wilmington, DE, USA). Detailed procedure was described in Supplementary Method.

### Turnaround time

The turnaround time was defined as the period from the time of sample registration until reporting by the Idylla^™^ *KRAS* Mutation Test or the Idylla^™^ *NRAS*-*BRAF* Mutation Test. Time is shown in working days assuming a Monday to Friday working week.

### Statistical analysis

Kappa statistics were used to compare the results of the Idylla^™^ *KRAS* and *NRAS*-*BRAF* Mutation Tests with the results of the MEBGEN RASKET^™^-B kit. Categorical variables were compared using the Fisher exact test. Continuous variables were compared between groups with the Mann–Whitney U test. All the statistical analyses were performed using JMP software, version 14.2 (SAS Institute Japan, Tokyo, Japan), and Prism software, version 8.4 (GraphPad Software, San Diego, CA, USA). A *p* value of < 0.05 was considered statistically significant.

## Results

### Study population

First, FFPE tissue samples from all 275 patients with colorectal cancer were retrieved for use in this study. All the samples were histologically confirmed as malignant CRC by pathologists. The clinical and pathological characteristics are summarized in Table 2. There were 145 (52. 7%) males and 130 females (47.3%) with a median age of 72 years (range: 32–92). The relative frequencies of histological type are 86.9% for intestinal type and 13.1% for the diffuse type. Tumor stage was 12.7% for stage 1, 34.2% for stage II, 45.5% for stage III, and 7.6% for stage IV. Among the 275 clinical specimens, 253 samples were subjected to both assays (Fig. 1). These include 28 FFPE samples with known *BRAF* mutations obtained by prescreening. The reasons for the exclusion of the 22 samples were the absence of tumor content or inappropriate tumor proportions (21 cases) as determined using hematoxylin and eosin (H&E) staining; the remaining specimen was judged to be unsuitable for inclusion by the pathologists because of demineralization.

**Table 2:** Baseline demographic and clinical characteristics for participants with test results

Variable	Retrieved cases (*n* = 275)	Mutation testing (*n* = 253)
	*n* (%)	*n* (%)
Sex		
Male	145 (52.7)	137 (54.2)
Female	130 (47.3)	116 (45.8)
Age		
Median (range)	72 y (32–92)	72 y (32–92)
Histological type		
pap/tub	239 (86.9)	222 (87.7)
por/muc/sig	36 (13.1)	31 (12.3)
TNM stage ^a^		
I	35 (12.7)	33 (13.0)
II	94 (34.2)	87 (34.4)
III	125 (45.5)	114 (45.1)
IV	21 (7.6)	19 (7.5)

*pap* papillary, *tub* tubular adenocarcinoma, *por* poorly differentiated adenocarcinoma, *muc* mucinous adenocarcinoma, *sig* signet-ring cell carcinoma

^a^According to the Japanese classification of colorectal, appendiceal, and anal carcinoma

### Mutations detected using the Idylla^™^*KRAS* and *NRAS-BRAF* mutation tests

**Fig. 2:**
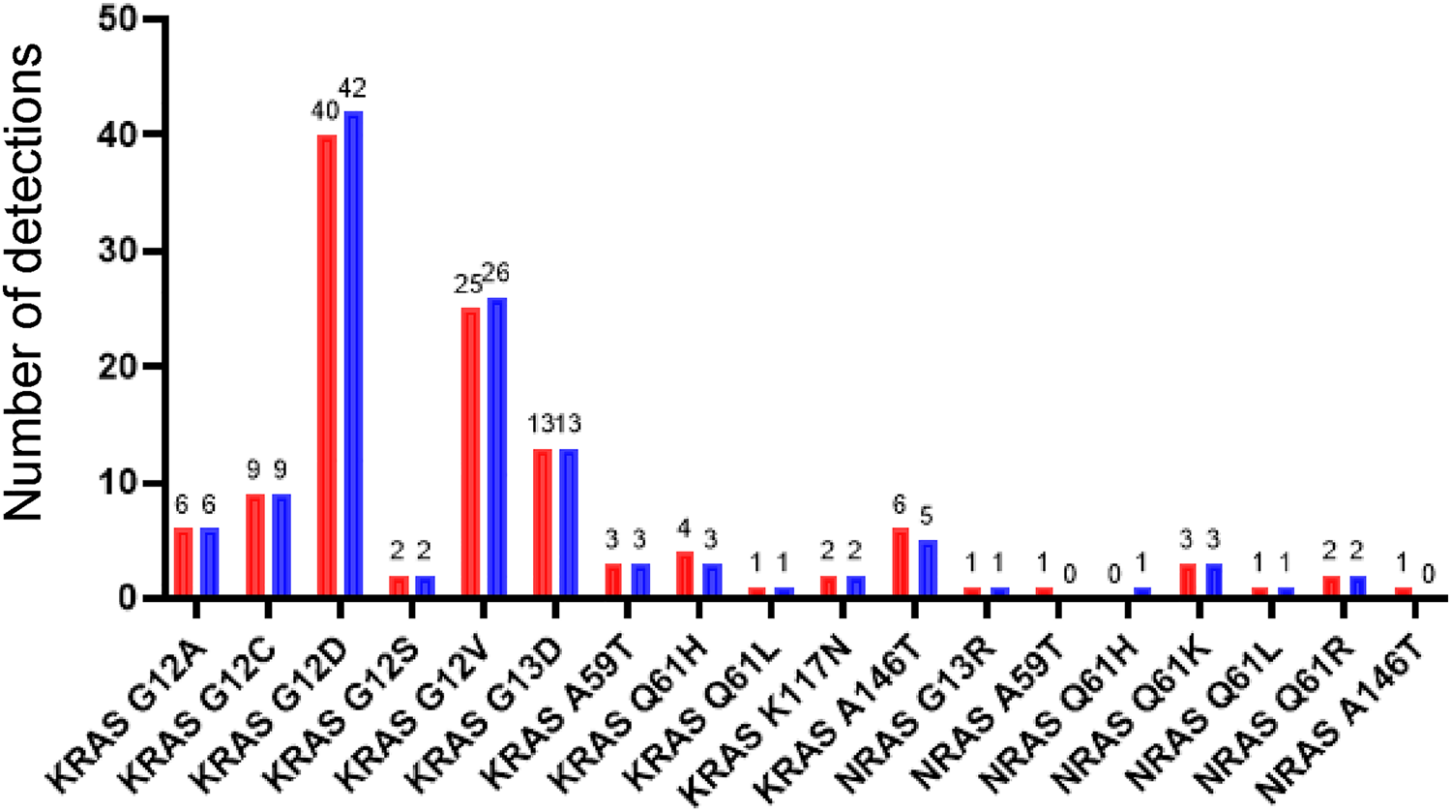
Mutations in *KRAS*, *NRAS*, and *BRAF* detected using the Idylla^™^ *KRAS* and *NRAS*-*BRAF* Mutation Tests (red bar) and the MEBGEN RASKET^™^-B kit (blue bar) in 253 FFPE specimens

The success rate for obtaining a valid mutational data without retest of the Idylla^™^ *KRAS* and *NRAS-BRAF* Mutation Tests was 97.6% (247/253). Among the 253 clinical specimens that were examined, 111 *KRAS* mutations (43.9%) and 9 *NRAS* (3.6%), and 36 *BRAF* mutations (14.2%) mutations were detected using the Idylla^™^ *KRAS* and *NRAS-BRAF* Mutation Tests (Fig. 2; details in Table S1). The G12D, G12V, G13D, and G12C *KRAS* mutations were the most frequently detected (40/111, 36.0%; 25/111, 22.5%, 13/111, 11.7%; and 9/111, 8.1%, respectively). *BRAF* mutations were detected in 36 specimens (including 28 *BRAF* mutation positive cases out of 208 prescreened cases) using the Idylla^™^ *NRAS-BRAF* Mutation Test. Therefore, *BRAF* frequency under spontaneous conditions is 17.8% (8/45).

*KRAS* mutation was associated with female sex (*p* = 0.0002). *BRAF* mutation positive cases were older age (*p* = 0.0198) and showed a higher frequency of poorly differentiated cancers (*p* < 0.0001) [19]. No differences in age, sex, histological type, or disease stage were observed between samples with and those without a *NRAS* mutation (Table S2).

### Method correlation agreement analysis

The Idylla^™^ results for the 253 cases that were examined were compared with the results of the MEBGEN RASKET^™^-B kit. The number of invalid tests for Idylla^™^ *KRAS* Mutation Test, Idylla^™^ *NRAS-BRAF* Mutation Test, and MEBGEN RASKET^™^-B kit were 7, 6, and 4, respectively. Discordances between the results of the Idylla^™^ *KRAS* and *NRAS*-*BRAF* Mutation Tests and those of the MEBGEN RASKET^™^-B kit were observed for four specimens. The positive agreement rate was 97.4% (151/155), the negative agreement rate was 95.7% (90/94), and the overall agreement rate was 95.3% (*κ* = 0.919, 95% CI 0.871–0.967) (Table 3).

**Table 3:** Comparison of Idylla^™^ *KRAS* and *NRAS*-*BRAF* mutation tests and MEBGEN RASKET^™^-B kit results for archived FFPE tissues

Idylla^™^	MEBGEN RASKET™-B					
	Positive			Negative	Invalid	Total
	*KRAS*	*NRAS*	*BRAF*	WT		
Positive						
* KRAS*	109	0	0	1	1	111
* NRAS*	0	7	0	2	0	9
* BRAF*	0	0	35	0	1	36
Negative						
WT	0	1	0	90	0	91
Invalid	3	0	0	1	2	6
Total	112	8	35	94	4	253

### Retests

A retest of 17 specimens (13 invalid and 4 discordant specimens) was performed using the Idylla^™^ *KRAS* and *NRAS*-*BRAF* Mutation Tests. Five of the eight specimens that were retested using the Idylla^™^ *KRAS* Mutation Test remained invalid. The result for one case changed from invalid to positive, one case changed from invalid to negative, and one case changed from positive to negative for *KRAS* mutation. Meanwhile, 2 of the 9 specimens that were retested using the Idylla^™^ *NRAS*-*BRAF* Mutation Test remained invalid. The results for two cases changed from positive to negative for *NRAS* mutation. Four invalid cases were validated (positive or negative) by retests. The four samples retested using the MEBGEN RASKET^™^-B kit showed that one case remained invalid, and three cases changed from invalid to positive.

### Sequencing analysis for cases with discrepancies

Amplicon sequencing was performed for the four cases with discrepant results between the Idylla^™^ *KRAS* and *NRAS*-*BRAF* Mutation Tests and the MEBGEN RASKET^™^-B kit. The gene mutation results detected by sequencing were consistent with the results detected using the MEBGEN RASKET^™^-B kit. No mutation was detected by the deep sequencing in three cases (K-N-001-*NRAS* A146T, K-N-151-*NRAS* A59T, and K-N-221-*KRAS* Q61H), in which mutations had been detected using the Idylla^™^ *KRAS* and *NRAS*-*BRAF* Mutation Tests (Table S3). Sequencing analysis identified *NRAS* Q61H mutations with a variant allele frequency of 22.6% in one case (K-N-214), in which no mutation had been detected using the Idylla^™^ *KRAS* and *NRAS*-*BRAF* Mutation Tests. This discrepancy is due to the difference in detectable sites between Idylla^™^ and RASKET. Idylla^™^ was only designed by *NRAS* Q61H (c.183A>C), whereas RASKET is designed to detect *NRAS* Q61H (c.183A>C and c.183A>T). K-N-214 was not detected by Idylla^™^ because it harbored the *NRAS* Q61H mutation of c.183A>T nucleotide base substitution.

### Turnaround time

The TAT is the time interval between the specimens received in the laboratory to the time of reports of the assay. The average TAT of the Idylla^™^ *KRAS* and *NRAS*-*BRAF* Mutation Test was 5.61 ± 1.99 working days (range 3–11 days). The TAT of the MEBGEN RASKET^™^-B kit was not determined because the testing was outsourced.

## Discussion

In this study, the success rate of the Idylla tests was 97.6%. This rate was considered a clinically acceptable level. However, the number of tissue specimens submitted was 275, and 22 specimens were excluded at the pre-analytical stage. Appropriate specimen preparation at the pre-analytical stage was considered important for assay success. Highly concordant results were obtained between the Idylla^™^ *KRAS* and *NRAS*-*BRAF* Mutation Tests and the MEBGEN RASKET^™^-B kit. The Idylla tests were shown to be reliable with a high positive concordance rate and negative concordance rate relative to the results of the MEBGEN RASKET^™^-B kit. Deep sequencing of four discordant cases detected no *RAS-BRAF* mutations in three cases. These results suggest that the mutations detected using the Idylla^™^ *KRAS* and *NRAS*-*BRAF* Mutation Tests were likely to be false-positive. This number of false-positive cases was considered clinically acceptable.

*KRAS* and *NRAS* mutations are known to be the most frequent actionable mutations observed in colorectal cancer. In the present study, the Idylla tests detected 111 *KRAS* mutations (43.9%) and 9 *NRAS* mutations (3.6%) in 253 specimens. Among the *KRAS* mutations, mutations in codons 12 and 13 of *KRAS* were detected at a high frequency. The frequencies of *KRAS* and *NRAS* mutations were similar to those in previous reports [20, 21]. We also detected *BRAF* V600E mutations. Mutations in the *BRAF* gene occur in approximately 12% of mCRC patients, with reported frequencies ranging from as low as 5% to as high as 21% [21–24]. The majority of *BRAF* mutations are V600E substitutions [25]. In our sample cohort, *BRAF* V600E mutations were detected in 36 samples, including 28 samples with known mutations as a result of prescreening. Therefore, eight additional *BRAF* V600E mutations were detected in 45 naïve specimens (17.8%). The design of the present study did not allow for an accurate estimation of the *BRAF* mutation frequency. Although the Idylla^™^ tests were not designed to detect other genotypes of *BRAF* mutations, the mutation frequencies for such genotypes are very low, and the present performance was considered to be clinically acceptable. When differences among histological types were examined, the *BRAF* V600E mutation was detected at a significantly higher frequency in poorly differentiated adenocarcinomas, compared with other histological types (*p* < 0.0001). These results were consistent with those of a previous paper reported by Yokota et al*.* They also reported that *BRAF* V600E mutations were frequently observed in mucinous adenocarcinoma of the colon in Japanese patients [19], but no *BRAF* mutations were observed in the mucinous types in our sample cohort. Further study might be necessary.

The TAT of the Idylla tests was 5.6 days, and no difference was seen between the Idylla^™^ *KRAS* Mutation Test and the Idylla^™^ *NRAS*-*BRAF* Mutation Test. Therefore, no time loss occurred when both kits were used together. The TAT of 5.6 days was caused by the working period for the preparation of the tumor tissue at the pre-analytical stage, including the sectioning of the samples and the confirmation of the tumor contents using hematoxylin and eosin staining. After sample preparation, there were hands-on in required for the Idylla tests were less than 2 min, excluding the nucleic acid extraction procedure; the time required from sample application until the end result was approximately 2 h. Therefore, the Idylla^™^ platform is expected to shorten the TAT in real-world clinical practice. In addition, since only one slide is necessary per Idylla test, it is clinically advantageous that only two slides for two tests are required.

In this study, we examined the clinical performance of the Idylla^™^ platform and found that its performance was comparable to that described in previous overseas reports [7, 26, 27]. This formal clinical performance study supports the IVD approval of this test in Japan. Currently, an Idylla^™^ system for circulating tumor DNA in blood samples have already been developed for *BRAF*, *KRAS*, *NRAS*, and *EGFR *[27, 28]. Liquid biopsy tests are also available using the same assay system as tumor tissue samples. To promote liquid biopsy testing in Japan, formal clinical performance tests are warranted. The usefulness of the Idylla^™^ system is good news for Japanese colorectal cancer patients, but the system might also be applicable to other types of cancer, such as non-small cell lung cancer.

In conclusion, the Idylla^™^ *KRAS* and *NRAS-BRAF* Mutation Tests for colorectal patients are reliable, simple, and rapid sample-to-result solutions for the detection of clinically important *KRAS*, *NRAS*, and *BRAF* mutations without the need for molecular expertise or infrastructure.

## Supplementary Information

Below is the link to the electronic supplementary material.

Supplementary file1 (DOCX 17 KB)

Supplementary file2 (XLSX 11 KB)

Supplementary file3 (XLSX 21 KB)

Supplementary file4 (DOCX 19 KB)

## Abbreviations

95% CI: 95% Confidence interval
CRC: Colorectal carcinoma
EGFR: Epidermal growth factor receptor
FFPE: Formalin-fixed paraffin-embedded
H&E: Hematoxylin and eosin
IUO: Investigational use only
IVD: In vitro diagnostics
TAT: Turnaround time
WT: Wild-type
