# Supplementary material for: Performance of Idylla™ RAS-BRAF mutation test for formalin-fixed paraffin-embedded tissues of colorectal cancer
Source: Int J Clin Oncol. 2022 Apr 26;27(7):1180–7. doi: 10.1007/s10147-022-02167-z (PMC9209352; doi:10.1007/s10147-022-02167-z)
Supplement: Supplementary file 1 — Supplementary file1 (DOCX 17 KB) [file 10147_2022_2167_MOESM1_ESM.docx]

**Supplementary Methods**

Idylla^TM^ *KRAS* and *NRAS*-*BRAF* Mutation Tests

The Idylla molecular diagnostic platform is a fully automated, allele-specific real-time PCR-based molecular diagnostic system. It combines sample preparation with PCR thermocycling to detect molecular targets from a variety of solid and liquid samples including FFPE slices. Briefly, FFPE tissue sections are placed directly into the Idylla^TM^ system cartridge, with no preprocessing. The cartridge is then inserted into the Idylla^TM^ platform, where a combination of high-intensity focused ultrasound technology, buffers, reagents and heat induces deparaffinization, tissue disruption, cell lysis and the subsequent release of nucleic acids. Allele-specific primers and fluorescent probes then perform real-time PCR amplification and mutation detection. All required consumables are provided in the cartridge. The Idylla technique uses microfluidics processing with all reagents on board for a single 5-μm slice. The operator is required to install a specific cartridge in which the macro-dissected tissue has been placed. The remaining process, including nucleic acid extraction, is fully automated. The results are ready in approximately 130 min for *KRAS* mutation and 115 min for *NRAS*-*BRAF* mutation, are presented on a screen, and can be printed.

Using Idylla^TM^ software, a set of parameters describing the generated PCR curves, such as the ΔCq value (calculated as the difference between the quantification cycle value [Cq] of the gene control signal and the Cq of the mutant signal) can be obtained. A sample is classified as mutation positive if the parameters of the generated PCR curve are within the validated range. Otherwise, the sample is reported as being mutation negative (i.e., wild-type [WT]). Of note, the Idylla^TM^ mutation tests do not report double mutants; in such cases, only the mutation with the smallest ΔCq is called. All samples with a valid wild-type signal but a ΔCq value outside the validated range are characterized as “no mutation detected.” The results were presented as “no mutation detected”, “mutation detected in gene X (*KRAS*, *NRAS* or *BRAF*) codon XX” or “invalid.” The *KRAS* mutation status was determined using the Idylla^TM^ *KRAS* Mutation Test, while the *NRAS* and *BRAF* mutations were determined using the Idylla^TM^ *NRAS*-*BRAF* Mutation Test, which is a separate cartridge.

MEBGEN RASKET^TM^-B kit

The MEBGEN RASKET^TM^-B kit include the mutation of *KRAS* codon 12 (G12S, G12C, G12R, G12D, G12V, and G12A), *KRAS* codon 13 (G13S, G13C, G13R, G13D, G13V, and G13A), *KRAS* codon 59 (A59T and A59G), *KRAS* codon 61 (Q61K, Q61E, Q61L, Q61P, Q61R, and Q61H), *KRAS* codon 117 (K117N), *KRAS* codon 146 (A146T, A146P, and A146V), *NRAS* codon 12 (G12S, G12C, G12R, G12D, G12V, and G12A), *NRAS* codon 13 (G13S, G13C, G13R, G13D, G13V, and G13A), *NRAS* codon 59 (A59T and A59G), *NRAS* codon 61 (Q61K, Q61E, Q61L, Q61P, Q61R, and Q61H), *NRAS* codon 117 (K117N), *NRAS* codon 146 (A146T, A146P, and A146V), and *BRAF* codon 600 (V600E).

Sequencing analysis

The Ion AmpliSeq Colon and Lung Cancer Panel (Thermo Fisher Scientific), which covers hotspots of 22 genes involved in colon and lung cancer. The Ion Xpress Barcode Adapters (Thermo Fisher Scientific) were ligated into the PCR products and were purified using Agencourt AMPure XP beads (Beckman Coulter). The purified libraries were pooled and then sequenced using an Ion Torrent S5 instrument and an Ion 550 Chip Kit (all from Thermo Fisher Scientific). DNA sequencing data were accessed through the Torrent Suite ver. 5.12 program (Thermo Fisher Scientific). Reads were aligned against the hg19 human reference genome, and variants were called using Variant Caller ver. 5.12. Raw variant calls were filtered with a quality score of <100 and were manually checked with the integrative genomics viewer (IGV; Broad Institute). Germline mutations were excluded with the use of the Human Genetic Variation Database (http://www.genome.med.kyoto-u.ac.jp/SnpDB).
