# Supplementary material for: Performance of Idylla™ RAS-BRAF mutation test for formalin-fixed paraffin-embedded tissues of colorectal cancer
Source: Int J Clin Oncol. 2022 Apr 26;27(7):1180–7. doi: 10.1007/s10147-022-02167-z (PMC9209352; doi:10.1007/s10147-022-02167-z)
Supplement: Supplementary file 4 — Supplementary file4 (DOCX 19 KB) [file 10147_2022_2167_MOESM4_ESM.docx]

**Table S2.** Clinicopathological features associated with *KRAS*, *NRAS*, and *BRAF* mutations.

| Gene | Variables |  | MT | WT | *p* |
| --- | --- | --- | --- | --- | --- |
| *KRAS* | Sex | male/female | 45/66 (40.5/59.5) | 88/47 (65.2/34.8) | 0.0002* |
|  | Age | median (range) | 71 (32-91) | 73 (33-92) | 0.0900 |
|  | Histological type | pap, tub/por, muc, sig | 100/11 (90.1/9.9) | 115/20 (85.2/14.8) | 0.3346 |
|  | TNM Stage ^a^ | I/II/III/IV | 17/35/52/7 (15.3/31.5/46.8/6.3) | 16/52/55/12 (11.9/38.5/40.7/8.9) | 0.4939 |
| *NRAS* | Sex | male/female | 6/3 (66.7/33.3) | 127/111 (53.4/46.6) | 0.5117 |
|  | Age | median (range) | 74 (64-85) | 72 (32-92) | 0.7681 |
|  | Histological type | pap, tub/por, muc, sig | 7/2 (77.8/22.2) | 209/29 (87.8/12.2) | 0.3146 |
|  | TNM Stage ^a^ | I/II/III/IV | 3/2/4/0 (33.3/22.2/44.4/0) | 30/85/104/19 (12.6/35.7/43.7/8.0) | 0.3293 |
| *BRAF* | Sex | male/female | 20/16 (55.6/44.4) | 113/98 (53.6/46.4) | 0.8583 |
|  | Age | median (range) | 76 (33-92) | 72 (32-91) | 0.0198* |
|  | Histological type | pap, tub/por, muc, sig | 20/16 (55.6/44.4) | 196/15 (92.9/7.1) | <0.0001* |
|  | TNM Stage ^a^ | I/II/III/IV | 3/14/15/4 (8.3/38.9/41.7/11.1) | 30/73/93/15 (14.2/34.6/44.1/7.1) | 0.6154 |

The asterisk indicates statistical significance. pap; papillary, tub; tubular adenocarcinoma, por; poorly differentiated adenocarcinoma, muc; mucinous adenocarcinoma, sig; signet-ring cell carcinoma, MT; mutant, WT; wild-type.

^a^ According to the Japanese Classification of Colorectal, Appendiceal, and Anal Carcinoma.
